# Molecular Interactions of Selective Agonists and Antagonists with the Retinoic Acid Receptor γ

**DOI:** 10.3390/ijms25126568

**Published:** 2024-06-14

**Authors:** Katarzyna Powała, Teresa Żołek, Geoffrey Brown, Andrzej Kutner

**Affiliations:** 1 Department of Organic and Physical Chemistry, Faculty of Pharmacy, Medical University of Warsaw, 1 Banacha, 02-097 Warsaw, Poland; 2 School of Biomedical Sciences, Institute of Clinical Sciences, College of Medical and Dental Sciences, University of Birmingham, Edgbaston, Birmingham B15 2TT, UK; g.brown@bham.ac.uk; 3 Department of Drug Chemistry Pharmaceutical and Biomedical Analysis, Faculty of Pharmacy, Medical University of Warsaw, 1 Banacha, 02-097 Warsaw, Poland; andrzej.kutner@wum.edu.pl

**Keywords:** receptor RARγ, ATRA, cancer stem cells, retinoids, RARγ antagonists, AGN205728

## Abstract

All-*trans* retinoic acid (ATRA), the major active metabolite of all-*trans* retinol (vitamin A), is a key hormonal signaling molecule. In the adult organism, ATRA has a widespread influence on processes that are crucial to the growth and differentiation of cells and, in turn, the acquisition of mature cell functions. Therefore, there is considerable potential in the use of retinoids to treat diseases. ATRA binds to the retinoic acid receptors (RAR) which, as activated by ATRA, selectively regulate gene expression. There are three main RAR isoforms, RARα, RARβ, and RARγ. They each have a distinct role, for example, RARα and RARγ regulate myeloid progenitor cell differentiation and hematopoietic stem cell maintenance, respectively. Hence, targeting an isoform is crucial to developing retinoid-based therapeutics. In principle, this is exemplified when ATRA is used to treat acute promyelocytic leukemia (PML) and target RARα within PML-RARα oncogenic fusion protein. ATRA with arsenic trioxide has provided a cure for the once highly fatal leukemia. Recent in vitro and in vivo studies of RARγ have revealed the potential use of agonists and antagonists to treat diseases as diverse as cancer, heterotopic ossification, psoriasis, and acne. During the final drug development there may be a need to design newer compounds with added modifications to improve solubility, pharmacokinetics, or potency. At the same time, it is important to retain isotype specificity and activity. Examination of the molecular interactions between RARγ agonists and the ligand binding domain of RARγ has revealed aspects to ligand binding that are crucial to RARγ selectivity and compound activity and key to designing newer compounds.

## 1. Introduction

Many studies since the 1980s have highlighted the importance of vitamin A (all-*trans* retinol) to the patterning and generation of tissues during embryogenesis [1] and tissue regeneration in the adult organism regarding control of the behavior of stem/progenitor cells [2]. The influence of all-*trans* retinoic acid (ATRA), the most active metabolite of vitamin A, was unresolved until RARα was cloned in 1987 [3,4]. There are three main RAR isoforms, namely RARα, RARβ, and RARγ, that are encoded by different genes. RARs belong to the nuclear receptor superfamily of transcription factors and many studies have led to an understanding of their action. RARs form various heterodimers with the three retinoid X receptors (RXRα, RXRβ, and RXRγ) or homodimers. Both dimers bind to specific retinoic acid response elements (RAREs) within the promoters of target genes. Gene transcription is activated by the binding of ATRA to the RAR, and gene expression is suppressed in its absence [5].

An early finding was that ATRA was able to drive neutrophil maturation of the leukemic promyeloid cell line HL60 via activation of RARα [6]. Acute promyelocytic leukemia (APL) is a rare and highly fatal subtype of acute myeloid leukemia (M3). Chemotherapy achieved a 75% to 80% complete remission rate, a medium duration from 11 to 25 months, and a 35% to 45% cure rate. In the early 1990s, the use of ATRA, together with arsenic trioxide, turned APL from a highly fatal to a curable disease [7]. Many APL patients have the (15;17) (q24; q21) reciprocal translocation between the RARα and promyelocytic leukemia (PML) genes [8] and a few other cytogenetic effects, and the key to success was targeting the fusion RARα oncoprotein. ATRA has failed to provide effective differentiation therapy for other cancers [9].

The above provides undisputable evidence regarding the benefits of using retinoids to treat a specific disease. An agonist of RARα was more potent than ATRA in driving the differentiation of HL60 cells [10]. Therefore, agonism of RARα would be sufficient for differentiation therapy of APL. However, and to return to the fact that ATRA/RARs are central regulators of gene expression to cell behavior, there must be many prospects for the use of retinoids to treat specific diseases. This will certainly require RAR isoform selective retinoids. This is because there are severe side effects to the use of ATRA, including skin and mucous membrane inflammation, elevation of serum triglycerides, bone resorption, headaches, and hypothyroidism [11]. To regulate gene expression in a desired manner, there will also be the need to switch an RAR isoform either on or off.

In this review, we focus on RARγ, which plays a role in controlling the behavior of stem cells. RARγ is also an oncogene, and antagonism of RARγ, or all RARs, kills cancer cells [12,13,14]. First, we look at how selective agonists and antagonists of RARγ were designed and how RARγ agonists interact with RARγ, providing information to the rationale design of even newer analogues. We then examine the prospective clinical uses of RARγ agonists and antagonists.

## 2. RAR-Mediated Gene Regulation

This begins with the binding of RAR/RXR heterodimers to RAREs within the promoters of target genes [15,16,17]. Gene transcription is inhibited in the absence of ATRA due to the recruitment of corepressors (CoR), such as nuclear receptor corepressor (NCoR) and silencing mediator of retinoic acid and thyroid hormone receptor (SMRT), and histone deacetylase. Histone deacetylase catalyzes the removal of acetyl from lysine ε-amino groups [18,19] to increase the positive charge by protonating the ε-amino moiety. This leads to a stronger electrostatic interaction between histones and the negatively charged DNA. Hence, chromatin is more condensed, and transcription is inhibited.

ATRA binding to an RAR induces a conformational change that leads to CoR silencing [20,21]. Subsequently, coactivators (CoA), including steroid receptor coactivator (SRC) and CREB-binding protein (CBP), attach to the RAR surface [19,21,22]. Further factors are recruited by CoA as adaptors, including the histone acetylase (HAT) or histone methyltransferases, histone demethylases, and DNA-dependent ATPases [19,23]. HAT transfers the acetyl group from acetyl-CoA to the ε-amino group of lysine. This neutralizes its positive charge and increases its hydrophobicity, causing chromatin to decondense [21]. This allows the transcription machinery to attach to the promoters of RAR-responsive genes; finally, RNA polymerase II and other transcription factors bind to initiate mRNA transcription [5].

## 3. The Structure of RARs

**Figure 1:**
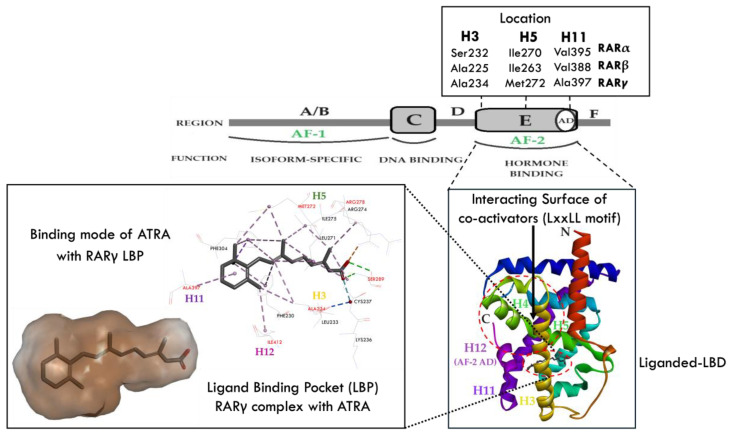
Domain structure of RARs. AF-1 = activation function 1; AF-2 = activation function 2; H3, H5, H11 = helices; LBP = ligand binding pocket; ATRA = all-trans retinoic acid; and LBD = ligand binding domain.

RARs have six functional domains, labeled A to F, from N terminal to C terminal (Figure 1) [5,24,25,26,27]. They include the A/B domain (also known as the AF-1 region), a C region DNA-binding domain (DBD), and an E region ligand-binding domain (LBD). The function of the F region, which is not present in all nuclear receptor superfamily members, is poorly understood.

A surface structure to the N-terminal A/B region of an RAR mediates interaction with CoA, CoR, and other transcription factors. The A/B region has a transcriptional activation function termed AF-1 [15] and is a target for post-translational modifications [5,28]. The C region DBD of an RAR binds to the RAREs within the promoters of target genes [24,25,26,27]. The DBD is composed of a core of 66 amino acid residues and formed by two modules containing Zn atoms, two α-helices, and extensions of the C-terminal fragment [5,28,29]. The DBD also plays a role in the formation of dimers with other receptors. The D region connects the DBD to the LBD, allowing the DBD to rotate so that the DBD and LBD can adopt different conformations without the risk of steric hindrance [20,24,30]. The D region is also involved in nuclear translocation and CoR binding [5]. The E region LBD of an RAR consists of a single protein domain, which is mainly organized in a helical scaffold called the α-helical antiparallel sandwich, containing 11 to 13 helices arranged in three layers [5,28]. It has four structurally distinct and functionally related surfaces. They are a dimerization surface that mediates interaction with partner LBDs, a ligand-binding pocket, a surface that binds CoA and CoR, and an activation function helix (AF-2) that mediates ligand-dependent transactivation [5,28].

The LBP is a hydrophobic pocket with a hole adjacent to the C-terminal α-helix H12. It is located behind helix H3 and in front of helixes H7 and H10. It is composed mainly of hydrophobic amino acids and a small number of polar amino acid residues (Lys236, Ser289, Arg278) at the end of the pocket. These amino acids play an essential role in the correct positioning of ligands. When a ligand binds to the receptor, a series of intracellular interactions cause a rearrangement of the receptor’s critical H12 helix. This closes the pocket leading to the formation of an external binding surface AF-2 which controls the ability of the ligand-bound RAR to activate transcription using the C-terminal helix of H12. It regulates the binding of CoA and CoR. The conformational flexibility of AF-2 plays a vital role in this process [28]. Keeping the LBP closed is critical for solid ligand binding and efficient transcriptional activity. The LBP of the RAR receptor is ‘’I’’ shaped, which makes it extremely sensitive to the size of the ligand. Compounds that are too long or too short are transcriptionally inactive. Specific residues, such as Phe230, Leu268, Leu271, Met272, Ile275, Phe304, Leu386, Ile389, and Ser390, play a crucial role in ligand binding [5,28]. The sequence and length of the F region are highly variable between the three RAR isotypes and extend from α-helix 12 (H12) to the C-terminus of the receptor. It is phosphorylated at several sites. The F region stabilizes the structure of the H12 helix in the absence of a ligand, thereby increasing CoR binding [5,15,20,24,28,31].

## 4. The Design of Synthetic RARγ Agonists and Antagonists

**Figure 2:**
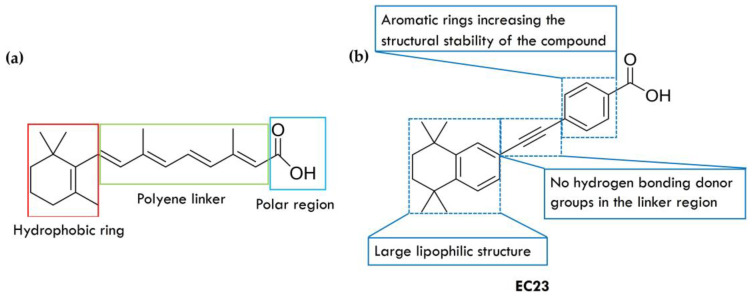
Chemical structure of ATRA (**a**) and synthetic retinoid EC23 (**b**).

ATRA is a potent agonist of all RARs. It consists of three parts: a hydrophobic part, a linker, and a polar part (Figure 2a) [32]. A large lipophilic structure forms the hydrophobic part of ATRA, which fills the hydrophobic space of the LBP entrance hole. The hydrophobic region of ATRA is formed by a β-ionone moiety connected to the extended methylated polyene linker. The linker region adds length and shape to ATRA. It also positions the polar region for binding to an RAR. At the end of ATRA is a polar region formed by a carboxylic group [33]. It binds at the end of LBP to the charged polar amino acid residues [11,34,35]. Structural studies have shown that ATRA and its two region isomers, 9-*cis*-RA and 13-*cis*-RA, adopt a wide range of low-energy conformations when binding to RARs. The polyene linker, which influences the biological activity of these compounds, is responsible for their high conformational flexibility.

Synthetic pan-RAR agonists mimic the shape and conformation of ATRA (Figure 2b). They have a linear structure and lack hydrogen bonding donor groups in the linker area. Removing the polyene structure found in natural retinoids and replacing it with aromatic rings is the most common approach to designing a pan-RAR agonist. This replacement increases the stability of the molecular structure, creating so-called arotinoids [36]. Replacing the flexible polyenes with more rigid structures allows the synthetic compound to mimic the most effective ATRA conformations [34].

Whilst the three RAR receptor isotypes share a high degree of structural similarity [15] there are essential differences between them. In RARγ, the H3 helix contains the amino acid residue Ala234, corresponding to Ser232 and Ala225 in RARα and RARβ. The H5 helix of RARγ contains Met272, corresponding to Ile270 and Ile263 in RARα and RARβ. In contrast, the Ala397 site in H11 of RARγ contains Val395 (RARα) and Val388 (RARβ). These differences result in slight variations in the size and architecture of the LBP of the RAR isotypes. The design of synthetic compounds that exhibit isoform selectivity exploits these variations [34,37].

**Figure 3:**
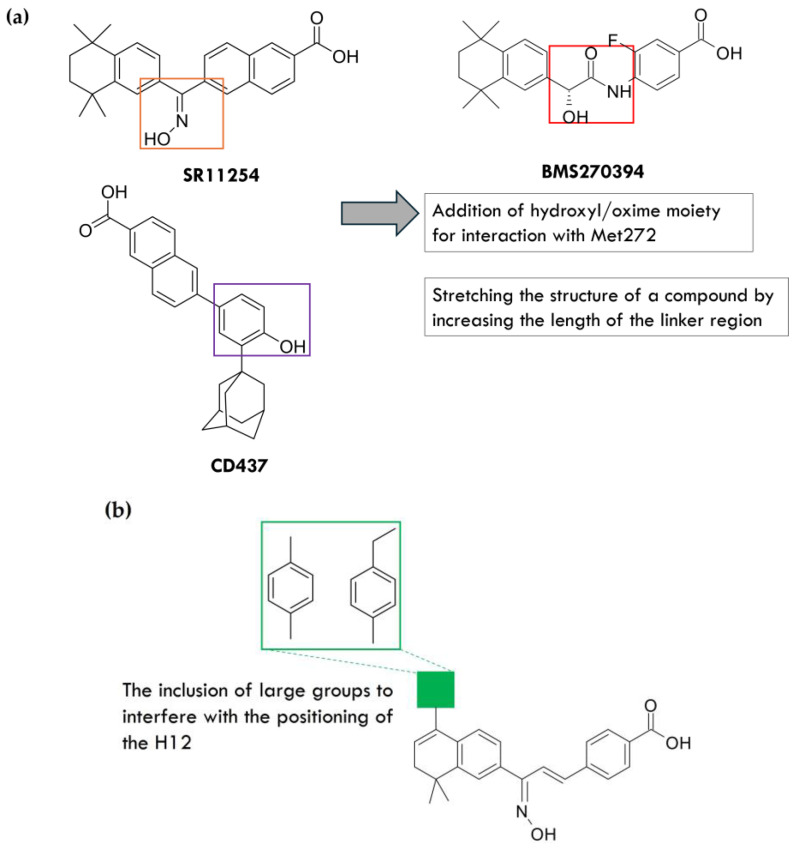
The design of a selective RARγ agonist (**a**) and antagonist (**b**).

Selective agonism of RARγ can be achieved by designing compounds that bind to the amino acid residues specific to this receptor, i.e., Ala234, Met272, and Ala397 in the LBP of RARγ. The amino acid Met272 is positioned roughly in the middle of the LBP, with the side chain situated above the linker region of a bound retinoid, and formation of a hydrogen bond with the Met272 side chain has been identified as a key strategy for increasing selectivity for RARγ. A hydroxyl or oxime moiety forms a sufficiently strong hydrogen bond with this amino acid moiety, and the compound’s structure must be stretched to form (Figure 3a). This stretching is obtained by increasing the length of the linker, e.g., by introducing a ketone. It will further reduce the compound’s affinity for RARα and increase the selectivity towards RARγ [38].

The same strategies used in the design of RARγ agonists were applied to the design of selective antagonists. Two approaches were used to achieve this goal (Figure 3b). They were the incorporation of large groups into the structure of the compound that would block the LBP-closure of the receptor by the H12 helix. This incorporation would lead to transcriptional inactivation. Additionally, ligands were designed that increased the affinity between the H12 helix and the CoR when bound to the receptor. This affinity prevents CoR removal and inhibits the binding of the CoA. It is known as reverse agonism [34]. For example, the pan-RAR antagonist AGN194310 contains an ethylbenzene-substituted thiochromene hydrophobic region. Upon binding to the receptor, the large phenyl substituent disrupts the positioning of the H12 helix, making AGN194310 a potent RAR antagonist [39]. It is possible to create compounds with receptor inverse agonist or antagonist activity by increasing the size of the groups in the hydrophobic region and subtly modulating the shape and orientation of other additional groups [34].

## 5. Crystallographic Structures of RARγ Agonist-Receptor Complexes

Over the last few years, the molecular basis of the interaction between synthetic analogs of retinoic acid and RARγ has been studied. The fundamental molecular interactions that are responsible for the isotype selectivity and biological activity of synthetic analogs have been investigated using molecular modeling techniques. The results have been compared with data obtained for natural RAR ligands [33]. These studies determined X-ray crystallographic structures of the LBDs of RARγ in complex with selective agonists. These provided further insight into the structure and properties of the complex that synthetic analogs form upon binding to RARγ. Analogs were positioned in the LBP, and their effects on its structure and activity were characterized. Knowledge of the molecular interactions of known ligands with RARγ is essential for the rational design of new molecules. The structure of selected complexes formed after docking agonists to RARγ have been described.

### 5.1. ATRA, a Nonselective RAR Agonist

**Figure 4:**
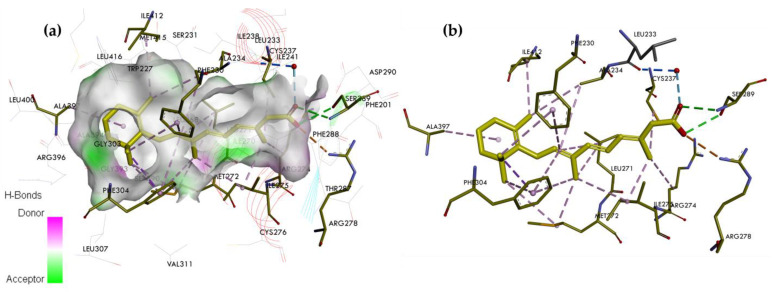
The crystal structure of the RARγ LBD complexed with ATRA. (**a**) Binding mode of ATRA in the binding pocket of RARγ. (**b**) Interaction site between ATRA and amino acids on the RARγ. In the skeleton model, the primary interactions responsible for selectivity are classical hydrogen bonds indicated with green and blue dashed lines, non-classical hydrogen bonds by grey dashed lines, and hydrophobic and electrostatic interactions by pink and orange dashed lines, respectively.

ATRA can bind to the three RAR isotypes in multiple conformations [33]. It has a similar affinity (ED_50_) for each isotype (RARα = 4 nM, RARβ = 5 nM, and RARγ = 2 nM) [32]. ATRA has been complexed with the RARγ, and the structure of the complex was solved by X-ray crystallography. The crystal structure of the RARγ LBD complexed with ATRA is available in the RCSB Protein Data Bank (ID: 2LBD—https://www.rcsb.org/structure/2LBD (accessed on 21 May 2024)) (Figure 4) [32]. 

The visualization shows that the non-polar region of ATRA forms favorable van der Waals interactions with the amino acid residues of the hydrophobic LBP of the RARγ, i.e., Leu268 on H5, Phe304 and Leu307 on H7, Arg396 and Leu400 on H11, and Ile412 and Met415 on H12 helix. The center of the pocket is narrower and predominantly hydrophobic. These pocket features allow it to interact well with the lipophilic polyene linker of ATRA. This region interacts with Ile275, Leu271, and Met272 on H5. The electrostatic attraction anchored the carboxylate to a small cluster of polar residues, Leu233, Arg278, and Ser289, at the base of the pocket. In the holo-conformation, H12 adopts a conformation that covers the entrance hole to the receptor pocket. The helix interacts with the hydrophobic regions of the retinoid. This interaction provides a stable platform for CoA attachment. The shape of ATRA has been slightly bent to fit into the LBP of RARγ [33,40].

### 5.2. Alitretinoin, or 9-cis-RA, a Non-Selective RARγ Agonist

**Figure 5:**
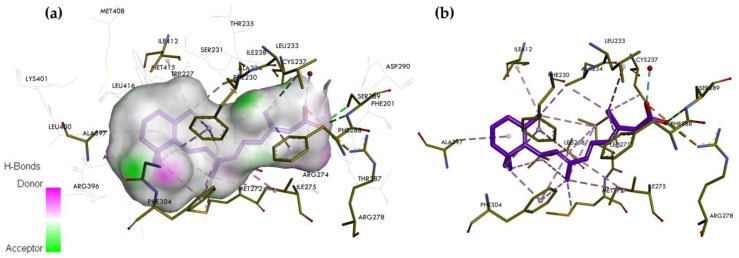
The crystal structure of the RARγ LBD complexed with 9-*cis*-RA. (**a**) Binding mode of 9-*cis*-RA in the binding pocket of RARγ. (**b**) Interaction site between 9-*cis*-RA and amino acids on the RARγ. In the skeleton model, the primary interactions responsible for selectivity are classical hydrogen bonds indicated with green and blue dashed lines, non-classical hydrogen bonds by grey dashed lines, and hydrophobic and electrostatic interactions by pink and orange dashed lines, respectively.

The LBD crystal structure of RARγ complexed with the agonist 9-*cis*-RA has been solved. This structure has allowed investigators to elucidate the molecular interactions between the ligand and the RARγ and explain the activity of the 9-*cis*-RA. (RCSB PDB ID: 3LBD—https://www.rcsb.org/structure/3LBD (accessed on 21 May 2024)) (Figure 5) [35]. This 9-*cis*-RA exhibited several low-energy conformations in the range of approximately 3–5 kcal. This range indicates the structure’s high flexibility, allowing it to adopt different positions in the LBP. The 9-*cis*-RA binds to the LBP of RARγ like ATRA. The carboxyl group is hydrogen-bonded to amino acid residues Arg278 and Ser289, the carbonyl group of the main chain to Leu233, and a water molecule [35]. The β-ionone group is in a small cavity near Ile412 [35]. The only significant difference between the RARγ-LBD-9-*cis*-RA and ATRA complexes is the position of the sulfur atom of residue Met272. The 19-methyl of the 9-*cis*-RA repels this sulfur atom. The steric clash of the 19-methyl of 9-*cis*-RA with residue Met272 (Ile270 and Ile263 in RARα and RARβ) may be the reason for the lower affinity of this compound for the RARγ, compared to RARα and RARβ [5,32,35].

### 5.3. BMS270394, a Selective RARγ Agonist

**Figure 6:**
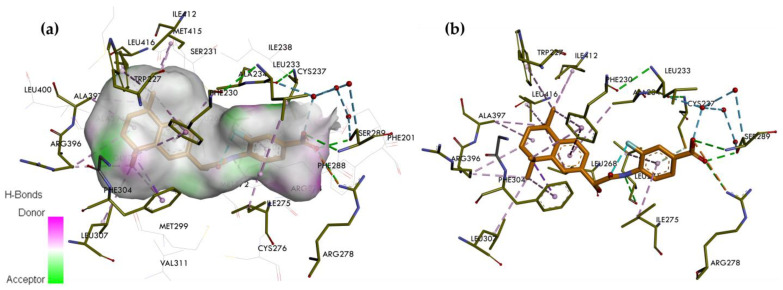
The crystal structure of RARγ LBD in complex with the selective RARγ agonist BMS270394. (**a**) Binding mode of BMS270394 in the binding pocket of RARγ. (**b**) Interaction site between BMS270394 and amino acids on the RARγ. In the skeleton model, the primary interactions responsible for selectivity are classical hydrogen bonds indicated with green and blue dashed lines, non-classical hydrogen bonds by grey dashed lines, and hydrophobic and electrostatic interactions by pink and orange dashed lines, respectively.

The synthesis and characterization of the crystal structure of the LBD of the RARγ complexed with the agonist BMS189961, a racemic mixture of the two enantiomers BMS270394 and BMS270395, showed that the *R*-enantiomer (BMS270394) is responsible for the biological activity of BMS189961, whereas the *S*-enantiomer (BMS270395) has no biological activity. A crystal structure of the LBD of RARγ in complex with BMS270394 (RCSB PDB ID: 1EX—https://www.rcsb.org/structure/1EXA (accessed on 21 May 2024)) [41] has been solved (Figure 6). Diffraction data provided detailed information on the structure of the enantiomers and their positioning in the RARγ LBP. Thus, the receptor’s structural discrimination of the enantiomers was discovered. BMS270395 adopts an energetically unfavorable conformation. In contrast, in the complex with RARγ, BMS270394 adopts a conformation like that of BMS189961. Hydrogen bonds are formed with polar residues by the carboxyl group of the ligands Arg278, Ser289, a water molecule, and Leu233 [35,38]. The fluorine atom forms short van der Waals interactions with Ala234, while the oxygen atom of the amide group forms short van der Waals interactions with Phe-230. Furthermore, the nitrogen of the amide group forms a hydrogen bond with the carbonyl group of amino acid residue Leu271. The position of the hydroxyl group of BMS270394 attached to the chiral center in the bridge linking 5,6,7,8-tetrahydro-5,5,8,8-tetramethyl-2-naphthalenyl and the benzoic moiety corresponds to the *R*-enantiomer. The basis for the selectivity towards RARγ is a hydrogen bond between the hydroxyl and the oxygen of BMS270394 with the sulfur of Met272. The distance between the oxygen of the hydroxyl group and the sulfur of Met272 is 3.20 Å. The small distance indicates high interaction strength. In addition, the steric positioning of the β-ionone side chains by Val395 and Val388 in RARα and RARβ reduces the ligand affinity for these receptors. Furthermore, the lack of affinity of BMS270394 for RARα is due to the steric hindrance formed by the Ser232 residue. The higher selectivity of BMS270394 towards RARγ is also due to the volume of the RARγ receptor binding pocket. This selectivity allows discrimination between RARγ and RARβ, which has a smaller binding pocket due to replacing the amino acid residue Ala397 with a bulkier valine [35,38]. The fluorine atom increases the selectivity of the compound towards the receptor, but only when attached to a hydroxyl [41].

### 5.4. CD437, a Selective RARγ Agonist

**Figure 7:**
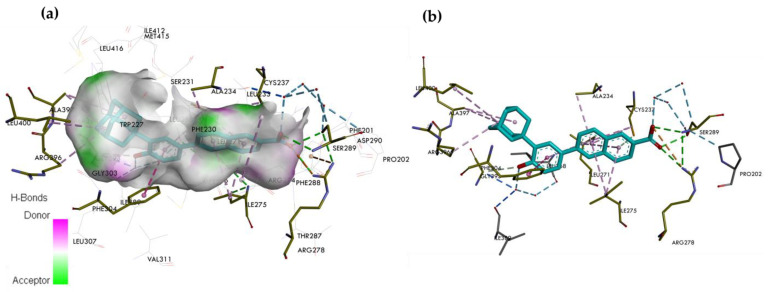
The crystal structure of RARγ LBD in complex with the selective RARγ agonist CD437. (**a**) Binding mode of CD437 in the binding pocket of RARγ. (**b**) Interaction site between CD437 and amino acids on the RARγ. In the skeleton model, the primary interactions responsible for selectivity are classical hydrogen bonds indicated with green and blue dashed lines, non-classical hydrogen bonds by grey dashed lines, and hydrophobic and electrostatic interactions by pink and orange dashed lines, respectively.

The crystal structure of the LBD of the RARγ complexed with the CD437 agonist is available from in RCSB PDB ID: 6FX0—https://www.rcsb.org/structure/6FX0 (accessed on 21 May 2024)) [42] (Figure 7). The CD437 agonist adopts a conformation in the RARγ binding pocket as observed for other RARγ agonists. The carboxyl group forms hydrogen bonds with Arg278, Ser289, and the backbone carbonyl group Leu233 via a water molecule. With minimal side chain disruption, the lipophilic adamantyl group is in the same position in the lipophilic pocket as the cyclohexene group of ATRA. The naphthyl ring overlaps well with ATRA’s polyene chain. The only difference was around the phenolic ring, which resulted in a shift of the Met272 residue. The flexibility of the Met-272 side chain and the interactions within the pocket created by this movement are responsible for the selectivity of CD437 towards RARγ. The other two isotypes, RARα and RARβ, have the more rigid isoleucine at the Met272 site. This isoleucine does not allow movement within the ligand-binding pocket. Displacing the Met272 side chain creates a small sub-pocket filled with two water molecules. The phenol ring of the CD437 molecule is in an ideal position to form a hydrogen bond with the first water molecule, which in turn bonds to the second water molecule. The two water molecules form hydrogen bonds as follows: the first to the carbonyl backbone of Ile-389; the second to the side chain of Ser390; and the carbonyl backbone of Leu268 [43].

### 5.5. SR11254, a Selective RARγ Agonist

**Figure 8:**
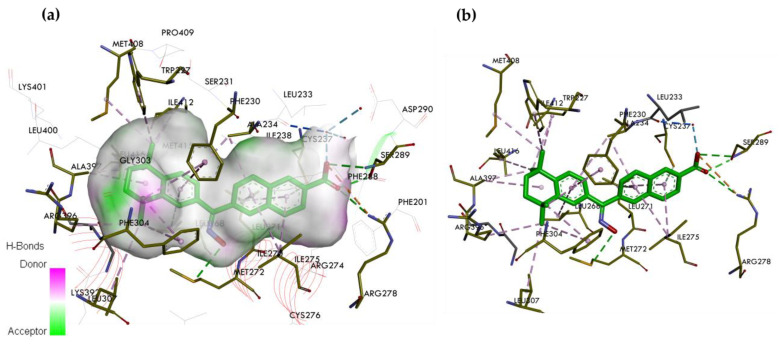
The crystal structure of RARγ LBD in complex with the selective RARγ agonist SR11254. (**a**) Binding mode of SR11254 in the binding pocket of RARγ. (**b**) Interaction site between SR11254 and amino acids on the RARγ. In the skeleton model, the primary interactions responsible for selectivity are classical hydrogen bonds indicated with green and blue dashed lines, non-classical hydrogen bonds by grey dashed lines, and hydrophobic and electrostatic interactions by pink and orange dashed lines, respectively.

The crystallographic structure of the LBD of RARγ complexed with SR11254 has also been solved (RCSB PDB ID: 1FD0—https://www.rcsb.org/structure/1FD0 (accessed on 21 May 2024)) [44] (Figure 8). SR11254 contains in its structure an oxime group attached to a bridge connecting the 5,6,7,8-tetrahydro-5,5,8,8-tetramethyl-2-naphthalenyl group and the 2-naphthalenic acid group. The replacement of the ketone by oxime results in the hydroxyl moving closer to Met272. The presence of this group favors the formation of favorable interactions with the Met272 side chain methyl. The oxime-derived hydroxyl confers isotype selectivity for RARγ by hydrogen bonding with the Met272 sulfur. SR11254 binds the receptor in two orientations of the oxime group, corresponding to the *Z*- and *E*-tautomers [38]. The carboxyl of SR11254 is anchored to the receptor by a network of hydrogen bonds formed with Arg278 and Ser289, a water molecule, and the carbonyl backbone of the Leu233 residue. Hydrogen bonds are between the hydroxyl of the *Z*- and *E*-tautomers and the sulfur of Met272. However, the bonds formed between the oxygen of the hydroxyl of the *Z*- and *E*-isomers and the methyls of Met272 and Ile275 are atypical. Bonding is stronger than van der Waals forces. This bonding indicates that hydrogen bonds exist between the oxygen of the hydroxyl and the hydrogens attached to the aliphatic carbons. These data suggest that the hydroxyl of both isomers of SR11254 acts simultaneously as a hydrogen bond donor and acceptor, resulting in a synergy between O-H---S and C-H---O hydrogen bonds [44]. Consideration of C-H---O hydrogen bonding in drug design is critical, especially when specific interactions are difficult to introduce in the hydrophobic environment of RARγ LBP [44].

In contrast to the known X-ray crystal structures of agonists bound to the RARγ, outlined above, the crystal structure of an antagonist complexed with RARγ has not been reported. Building up new knowledge on this missing molecular interaction is currently a significant challenge.

## 6. Targeting of Therapies to RARγ Agonism

### 6.1. AGN204647, a Selective RARγ Agonist

**Figure 9:**
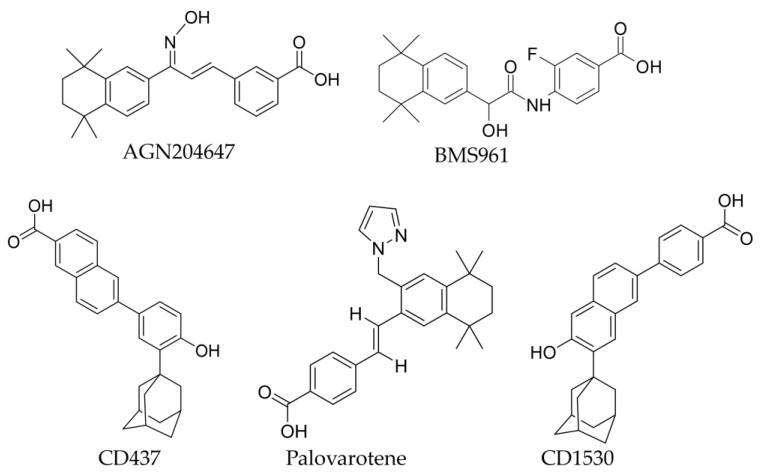
Structures of the RARγ agonists.

AGN204647 (Figure 9) is a potent RARγ agonist. The properties of this agonist have been investigated regarding heterotopic ossification (HO). The RARγ agonist was a potent inhibitor of heterotrophic ossification when administered intramuscularly and subcutaneously. It also blocked the formation of ectopic bone mass in transgenic mice carrying the active ALK2Q207D mutation. This blocking suggests that AGN204647 is effective in patients with fibrodysplasia ossificans progressive (FOP), which is a rare genetic disorder that causes significant disability and morbidity. When the RARγ agonist AGN204647 was applied to micro-mass cultures of embryonic limbal mesenchymal cells (E11.5), low levels of cartilaginous nodules were observed revealing that the compound inhibited chondrogenesis. At similar doses, AGN204647 had a more significant inhibitory effect on cells than ATRA. In mice treated with the RARγ agonist, the bone markers osteocalcin and tartrate-resistant alkaline phosphatase were undetectable [45]. The compounds showed minimal side effects during the study. There was no evidence of a rebound effect when the treatment was terminated. Only a short delay in repairing long bone fractures was observed in mice receiving the highest dose of AGN204647 [45].

### 6.2. CD1530, a Selective RARγ Agonist

The RARγ agonist CD1530 (Figure 9) has a high affinity for RARγ; the ED_50_ values obtained were for RARα = 2750 nM, RARβ = 1500 nM, and RARγ = 150 nM [11]. CD1530 inhibited chondrogenesis in mouse models and heterotopic ossification [45]. The activity of CD1530 was also tested in conjunction with the mutation in the bone morphogenetic protein receptor ALK2- ALK2R206H using mice that have a specific mutation in the ALK2 gene-ALK2Q207D. These mutations may be associated with fibrodysplasia ossificans progressiva (FOP). No ossification was observed in mice treated with CD1530, which suggests that CD1530 might be effective in patients with FOP. Whether RARγ agonists were effective against HO when treatment was delayed was investigated. For this purpose, CD1530 was administrated on day six, corresponding to HO’s chondrogenic phase. The RARγ agonist inhibited HO. The agonist was also given on day 12 during the osteogenic phase. In this case, the RARγ agonist had minimal or no effect. CD1530 had a minimal effect when a bone was already formed. Therefore, treatment should start as early as possible [45].

### 6.3. Palovarotene, a RARγ Agonist

Palovarotene (Figure 9) has the properties of a RARγ agonist. It inhibited chondrogenesis and HO in mice [45]. Palovarotene prevents HO in FOP patients by binding to RARγ, which inhibits bone morphogenetic protein and caenorhabditis elegans protein SMA (mother against decapentaplegic MAD; SMAD) 1/5/8 signaling. Disruption of these pathways prevented chondrogenesis and, ultimately, HO. This prevention enabled the repair and regeneration of muscle tissue. Palovarotene was the first agent to be approved for reducing HO formation in adults, children aged eight years and older, women and girls aged ten years or older, and older men with FOP [46].

### 6.4. CD437, a Selective RARγ Agonist

CD437 (Figure 9) agonism of RARγ led to the upregulation of genes associated with cell differentiation and induced apoptosis of tumor cells without affecting normal cells [47]. CD437 induced apoptosis in several cancer cell types independent of RARγ activation [48]. These included ovarian adenocarcinoma cell lines [49], hepatocellular carcinoma cells [50], human myeloma cells [51], human breast cancer cells, and human lung cancer cell lines [52]. CD437 induced apoptosis of LNCaP and PC-3 prostate cancer cell lines by arresting their cell cycle in the S phase [53]. It follows that CD437 can induce both cell differentiation and apoptosis. These findings make CD437 a good prototype for new drugs that may be clinically more effective than ones with just one type of activity [48]. The structure of the RARγ agonist CD437 and its binding to the receptor in the ligand binding pocket was used to design a drug candidate for treating acne. A new RARγ agonist CD5789 (trifarotene) has been identified [54]. Trifarotene combines the potency and selectivity of RARγ agonists. It received its first international approval for topical use to treat acne vulgaris in patients nine years and older [42,54,55].

### 6.5. BMS961, a RARγ Agonist

The ED_50_ value for BMS961 (Figure 9) for RARγ was 30 nM and BMS961 was investigated for its action on the epidermis. Topical treatment of mouse skin with BMS961 increased the number of proliferating keratinocytes in the epidermis. BMS961 also increased the expression levels of genes that regulate epidermis homeostasis, including the serine peptidase inhibitor Kazal type 5 (Spink5). BMS961 also promoted the differentiation of human esophageal epithelium from pluripotent stem cells but not normal progenitor cells [5,56].

## 7. Targeting of Therapies to RARγ Antagonism

There are two main rationales to the use of an RARγ antagonist to treat cancer. First, RARγ plays a key role in regulating the behavior of stem cells and, therefore, there is the prospect of controlling cancer stem cells (CSC). The activity of RARγ was required to sustain hematopoietic stem cells which were reduced in knockout mice [57]. Similarly, stem cell populations were maintained at the expense of bone and ganglion development from cranial neural crest stem cells, and fin development from mesodermal stem cells, when zebrafish embryos were treated with nM amounts of a RARγ agonist. Fin development was restored by agonist washout or the use of a RARγ antagonist [58]. Secondly, RARγ is an oncogene for several cancers, as evidenced by overexpression in human colorectal cancer, cholangiocarcinoma, hepatocellular cancer, ovarian cancer, pancreatic ductal adenocarcinoma, and renal cell cancer [12]. Overexpression was associated with increased cell proliferation, rapid disease progression, and a poor prognosis. Support for a role for RARγ in promoting the proliferation of cancer cells has been provided from knockdown and knockout studies [59]. APL cases have been described whereby the oncogenic fusion protein involves the RARγ gene in rearrangement as opposed to RARα [60] and patients with these translocations did not respond to ATRA treatment [61].

### 7.1. AGN205728, a Selective RARγ Antagonist

ATRA levels close to the detection limit were present in patients’ cells, whereas levels in surrounding normal tissue and benign prostate hyperplasia were up to eight times higher (see also below). This is important because RARγ is activated by nM concentrations of ATRA whereas 100-fold more is needed to activate RARα. Prostate cancer cells appear to have adapted to survive in low ATRA. Low concentrations of ATRA (10^−11^–10^−9^ M), sufficient to activate RARγ, stimulated the proliferation of the prostate cancer cell lines LNCaP, DU145, and PC-3, and the RARγ agonist AGN205327 exerted the same effect. For the three prostate cancer cell lines, treatment with 10^−10^ M ATRA increased the colony formation and the percentage of stem cell-like colonies [62].

**Figure 10:**
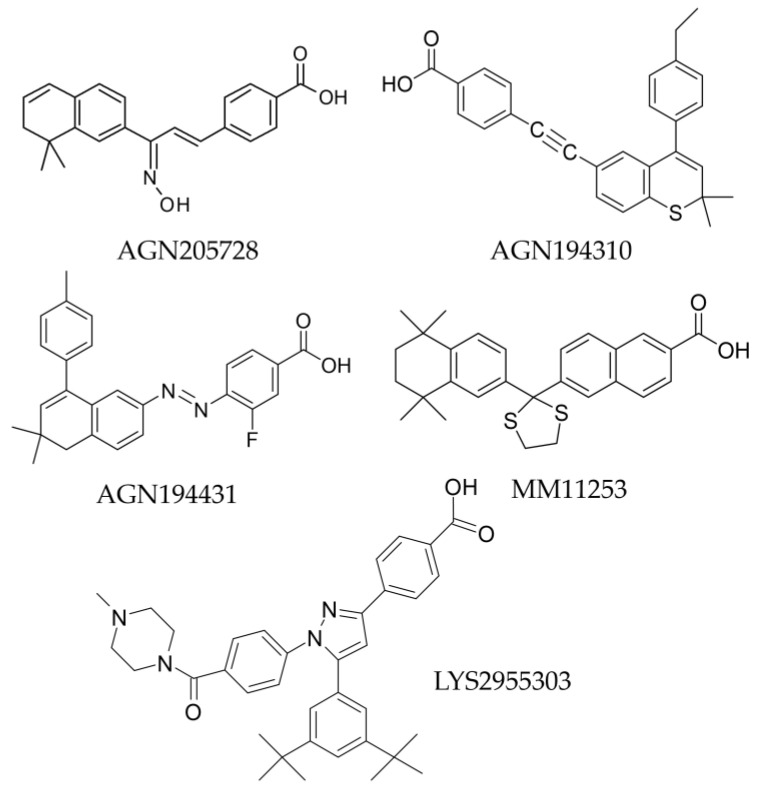
Structures of RARγ antagonists.

The RARγ antagonist AGN205728 (Figure 10) has a high affinity for RARγ with ED_50s_ values of RARα = 2400 nM, RARβ = 4248 nM, and RARγ = 3 nM [63]. AGN205728 was effective in driving the growth arrest and cell death of flask cultures of patients’ prostate cancer cells and the LNCaP, PC3, and DU145 cell lines [62]. AGN205728 prevented colony formation by the CSC-like cells of the prostate cancer cell line at a concentration of 5 nM (IC_50_), which is close to its ED_50_. It, therefore, targeted both CSC and non-CSC. For prostate cancer cell line cells, ATRA and the selective RARα antagonist AGN196996 were substantially less effective than AGN205728. Normal prostate epithelial cells and non-neoplastic RWPE-1 cells had a significantly reduced response to AGN205728. Treatment of cells with AGN205728 increased the number of cells in the G1 phase of the cell cycle, accompanied by a decrease in the number of cells in the S phase followed by cell death via necroptosis. A combination of docetaxel and a low dose of AGN205728 (10^−7^ M) showed a substantial reduction in prostate cancer cell viability, revealing that RARγ antagonists combined with chemotherapy may be effective in treating prostate cancer. Increasing the sensitivity of prostate cancer cells to taxemes and other therapeutic agents would reduce toxicity and the acquisition of resistance to these drugs [62].

### 7.2. AGN194310, a Pan-RAR Antagonist

AGN194310 is a pan-antagonist of RARs (Figure 10) that binds to RARα, RARβ, and RARγ receptors with equal and high affinities (ED_50s_ = 4.3, 5, and 2.5 nM, respectively) [63]. AGN194310 caused growth arrest and cell death of flask-grown DU145, LNCaP, and PC3 prostate cancer cells [14,64]. Antagonizing all RARs (AGN194310) was 12–22 times more potent than agonizing all RARs (ATRA) in driving the growth arrest of prostate cancer cells. Regarding the inhibition of colony formation by the cell line cells, the IC_50s_ were ~16 nM and close to the ED_50s_ for RARα, RARβ, and RARγ, confirming the potency of AGN194310. As seen for AGN205728, AGN194310 was effective against both CSC and non-CSC-like cells, normal prostate epithelial cells were less sensitive to AGN194310, and human peripheral blood lymphocytes and primary human fibroblasts were not affected [64]. Cell line cells treated with AGN194310 arrested growth in the G1 phase of the cell cycle, followed by death by necroptosis [64]. The nonselective RAR antagonist AGN94310 was also highly effective in killing pediatric patients’ primitive neuroectodermal and astrocytoma cancer cells, including the prevention of neurosphere formation by cancer stem cells [12].

### 7.3. AGN194431, a RARβ and RARγ Antagonist

AGN194431 (Figure 10) binds more strongly to RARβ receptor (ED_50_ = 6 nM) and RARγ (ED_50_ = 70 nM) than to RARα (ED_50_ = 300 nM). AGN194431 showed weaker activity than AGN194310 in inhibiting colony formation by all prostate cancer cell lines tested. The IC_50_ values obtained were 99 ± 10 nM for LNCaP cells, 104 ± 2 nM for PC3, and 88 ± 12 nM for DU145 cells [14].

### 7.4. MM11253, a Selective RARγ Antagonist

The ED_50_ values obtained for this antagonist were RARα = 1000, RARβ > 1000, and RARγ = 44. MM11253 (Figure 10) was tested in a study designed to demonstrate that activating RARγ-mediated transcription correlated with inhibition of 12-O-tetradecanoylphorbol-13-acetate (TPA) activity. TPA is the enzyme that induces ornithine carboxylase (ODC) activity. Increased levels of ODC were observed in tumors of 2C5 rat epithelial cells and the dorsal epidermis of CD-1 mice. A study of papilloma formation was conducted using a tumor initiation and promotion model in CD-1 mice. The effects of retinoids on the transcription factors c-Myc12 and ZBP-8913, which modulate ODC promoter activity, were also investigated. Significantly fewer tumors were observed in the study groups receiving high doses of the RARγ antagonist than in the control group. MM11253 at higher concentrations was an inhibitor of ODC ornithine carboxylase activity in 2C5 cells reducing the induction at 1 mM [65].

### 7.5. LYS2955303, a Selective RARγ Antagonist

The ED_50_ values obtained for LYS2955303 (Figure 10) for the different RAR isotypes were RARα > 1700 nM, RARβ > 2980 nM, and RARγ = 1.9 nM [66]. This compound was analyzed in studies to identify a potent RARγ receptor antagonist to treat osteoarthritis [66]. LYS2955303 showed good pharmacokinetic properties and effectively treated a model of MIA characterized by osteoarthritis-like joint pain. In addition, LYS2955303 had fewer side effects than RARα antagonist [66].

Cell line studies have examined the significance of high RARγ levels. RARγ is overexpressed in the HT29, HCT116, RKO, and SW480 colorectal cancer cell lines as compared to normal HCoEpiC colonic epithelial cells [67]. Overexpression was predominantly in the cytoplasm of the cancer cells, as seen also for the HEK293T kidney and HeLa cell lines [68]. RARγ knockdown increased the sensitivity of the HT29, HCT116, and RKO lines to 5-fluorouracil (5-FU), oxaliplatin, and vincristine. The investigators showed that RARγ overexpression contributed to chemoresistance by upregulating the expression of multidrug resistance 1 (MDR1), which occurred via activation of Wnt/β-catenin signaling, presumably by a non-genomic mechanism [67]. Overexpression of RARγ was observed in the cytoplasm of hepatocellular cancer tissues and the HepG2 cell line cells, and RARγ transfection promoted HepG2 colony formation in vitro and the growth of HepG2 xenografts in mice [69].

Yes-associated protein (YAP) is an oncogene and a CoA for RARγ. Elevated expression has been reported for bladder, cervical, colon, gastric, non-small cell lung, esophageal, and ovarian cancers [70]. For colon cancer, high expression correlated with disease relapse, metastasis, and poor survival post-5-FU treatment. Escape from 5-FU-mediated cell death has been attributed to cells entering a quiescent and stem-like state [71,72]. The YAP(S1127A) mutant protein (ser/ala substitution at 127) promotes YP nuclear localization and transcriptional activities [73]. Stable ectopic expression of YAP(S1127A) in HT29 and 5F31 colorectal cancer cell lines led to increased expression of mRNAs for RARγ and CYP26A1, a RARγ target gene [74], and these mRNAs were reduced in YAP-silenced cells. Nuclear YAP pulldown, coupled with mass spectrometry and chromatin immunoprecipitation (ChIP)/re-ChIP experiments, for 5-FU sensitive and 5-FU resistant HT29 cells, showed that YAP colocalized and interacted in the nucleus with RARγ and RXRs. YAP is, therefore, a *bona fide* RARγ CoA via RAREs. YAP activation leading to increased activity of RARγ reinforced stem cell traits within HT-29 cells, supports the view that 5-FU resistance relates, in part, to cells entering a stem-like state. Conversely, YAP silencing and the use of the pan-RAR antagonist BMS493 and vitamin A depletion, to prevent RAR-mediated signaling, downregulated stem cell traits of HT29 and 5F31 cells, including their capacity to renew [75]. For colon cancer cells, other investigators have argued that YAP synergizes with Wnt/β-catenin signaling [76]. Wnt/β-catenin signaling levels identify CSC [77], and secreted proteins that potentiate Wnt signaling are oncogenic drivers to colorectal cancer [78,79].

## 8. Targeting Inhibition of ATRA Synthesis to Treat Cancer

As above, antagonism of RARγ was highly effective in killing cancer stem cells. Another way to achieve this is to block endogenous ATRA synthesis, which is possible because some cancer cells live in an environment with access only to low levels of ATRA. Moreover, all-trans retinol uptake and/or metabolism are often disrupted in cancer cells, leading to reduced ATRA availability [80].

**Figure 11:**
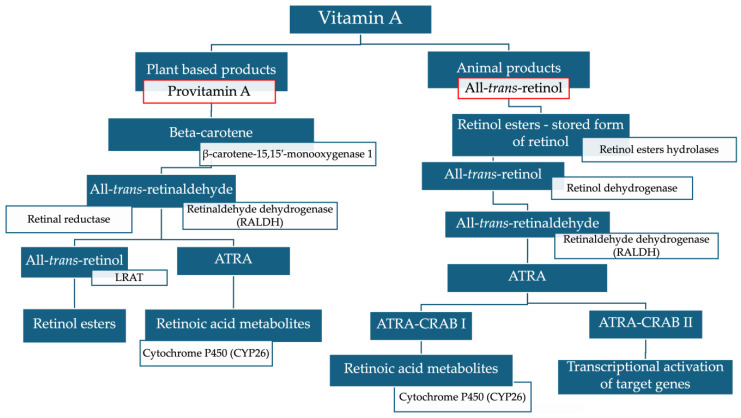
Scheme of all-*trans*-retinoic acid metabolism. ATRA = All–*trans*-retinoic acid; RALDH = retinaldehyde dehydrogenase; LRAT = lecithin retinol acyltransferase; CRAB I = cellular retinoic acid-binding protein I; CRABP II = cellular retinoic acid-binding protein II; CYP26 = cytochrome P450 26 enzyme.

All-trans-retinol obtained from the diet is stored as retinol esters in the body for release according to tissue needs [81,82]. Figure 11 shows ATRA synthesis from all-trans retinol. Aldehyde family members (ALDH) play a key role because they oxidize all-*trans*-retinaldehyde to ATRA. They have been reported to be absent and overexpressed in cancer cells, leading to abnormal ATRA levels. The low level of ALDH1A2 observed in head and neck squamous cell carcinoma cells was associated with a poor disease prognosis [83]. ALDH1A1 and ALDH1A2 mRNA enzymes were not detected in the LNCaP prostate cancer cell line, and low levels of ATRA have been reported for patients’ prostate cancer cells. ALDH1A3 mRNA was expressed in prostate cancer cells in response to androgens [84]. Histological studies have confirmed a lack of ALDH1A2 expression in patients’ prostate cancer cells compared to normal prostate epithelium [85].

ALDH1A1 and ALDH1A3 influence the phenotype of prostate cancer CSC, which are responsible for metastasis. ALDH1A1 positively regulates cancer cell survival, extravasation, and metastasis, while ALDH1A3 plays the opposite role. Prostate cancer progression is associated with increased interaction between ALDH1A1 and the androgen receptor (AR) and RAR. The activity of polo-like kinase 3 (PLK3) was also investigated. ALDH1A1 and ALDH1A3 expression regulated PLK3 in an AR- and RAR-dependent manner. PLK3 is involved in controlling prostate cancer cell proliferation, migration, DNA repair, and radio-resistance. An increase in ALDH1A1 in prostate cancer bone metastases was associated with high PLK3 expression. The results demonstrated that ALDH1A1 and PLK3 may serve as biomarkers to predict metastasis and the development of radio-resistance in prostate cancer patients and may represent potential therapeutic targets to eliminate metastatic cells [86].

ALDH1A1 overexpression has been observed in various cancers, including breast, colon, esophageal, liver, ovarian, pancreatic, and gastric [87], and the effect on disease prognosis varies between cancer types. The level of ALDH1A1 protein expression in breast cancer and its association with the treatment outcome was age-dependent [88]. ALDH1A1 expression increased the expression of DNA polymerase θ (Polθ, encoded by the POLQ gene) in ovarian cancer cells. ALDH1A1 promoted POLQ expression by activating ATRA signaling. Inhibition of ALDH1A1, using the pharmacological inhibitor NCT-505, in combination with the PARP inhibitor Olaparib, reduced the viability of patient-derived organoid cells. These cells carried BRCA1/2 mutations and showed positive expression of ALDH1A1 [89]. 

For MCF-7 breast cancer cells and colon cancer cells, increased expression of the Stra6 protein had impaired all-*trans*-retinol uptake and Stra6 was not detectable in healthy colon tissue [90,91]. LRAT is involved in the esterification of all-*trans*-retinol to retinol esters and the level of LRAT mRNA was lower in the PC-3 prostate cancer cell line than in normal prostate epithelial cells [91]. In this case, retinol cannot be adequately metabolized and stored, leading to ATRA deficiency [91]. Bladder, breast, kidney, oral, and skin cancers also have reduced LRAT expression and low intracellular levels of retinol esters [92]. CRABPII protein allows ATRA to enter the nucleus of cells and overexpression has been observed in breast, hepatocellular, and lung cancers. This impairs the transport of ATRA to the nucleus and, consequently, RAR activation [93]. By contrast, recent studies have shown that activated pancreatic cancer cells have lower levels of CRABPII protein expression than resting cells [94]. CRBP1 is a master regulator of retinol homeostasis in many tissues, including the liver, kidney, lung, brain, and hepatic stellate cells [95], and loss of CRBP1 expression has been observed in prostate cancer patients [96].

From the above findings, the use of isoform specific ALDH inhibitors has provided a promising anticancer treatment strategy. Inhibition of these enzymes, by 673A, DIMATE, DEAB, NCT-501, silybin, and solomargine, was effective against lung, ovarian, prostate, and uterine cancer cells [59]. Therapies based on ALDH enzyme inhibition have effectively eliminated the CSC cells of gynecological cancers [97].

## 9. Summary and Conclusions

ATRA has a widespread influence on processes that are crucial to cell growth, differentiation, and the acquisition of biological properties that are key to cell functions. There are three RARs, and RARα and RARγ have discrete physiological roles. For example, RARα and RARγ regulate myeloid progenitor cell differentiation and stem cell maintenance, respectively, and these different actions are important to balancing the production of mature cell types. Therefore, dysregulation of an individual RAR is most likely to be seen for diseases, for example, overexpression of RARγ in a wide range of cancer cells. Extending the therapeutic use of retinoids, therefore, necessitates the use of RAR isoform-selective agents. Highly selective agonists and antagonists of RARγ are available and studies to date have revealed that they have considerable potential as therapeutics for diseases as diverse as cancer, psoriasis, heterotopic ossification, and acne. For example, findings from in vitro studies have shown that the use of an RARγ antagonist kills cancer stem cells [97] and avoids the unwanted side effects of ATRA. In this case, targeting RARγ presents an effective avenue to the treatment of cancer.

When the RARγ agonist AGN205327 and RARγ antagonist AGN205728 were designed and synthesized, and for a good number of other RARγ selective agents, the RARγ crystal structure was not available. AGN205327 and AGN205728 were obtained by synthesizing retinoids with selective modifications together with the use of transactivation assays to test for RAR agonism and antagonism. To ensure RARγ selectivity, assays were undertaken using cell lines that had been transfected to overexpress either RARγ, RARα, or RARβ. For compounds undergoing final development to a drug, there may well be the need to modify to, for example, improve solubility or pharmacokinetics. Understanding the differences in molecular interactions between RAR agonists and antagonists and RARs is critical to designing structures that retain isotype specificity and potency and increasing such if needed.

The three-dimensional structures and crystallographic parameters of several agonists complex with RARγ LBDs are already deposited in crystallographic databases. Examination of the docking findings obtained for RARγ agonists has revealed key aspects of ligand structure that are important to the potent and selective agonism of RARγ. Compound AGN205728 is a highly selective RARγ antagonist. Its biological activity profile has been described, but there are currently no docking results from crystallographic studies for compounds that antagonize RARγ. What is not known is the three-dimensional structure of the RARγ AGN205728 complex and how RARγ and AGN205728 interact with each other. In this case, the design of new RARγ antagonists can now rely on docking studies of the conceived structures to the LBP of RARγ and calculations of the binding Gibbs free energy. It is hypothesized that the docking pose of a new antagonist and its interactions with the amino acid residues forming the hydrophobic end and the hydrophilic entrance of LBP should be like that of the known antagonist, and distinct from that of the agonists. Evaluating the biological activity of the new RARγ antagonists designed in this way will verify the hypothesis.

The findings from the examination of molecular interactions are important to the future rational design of drugs to be used to treat patients. The isoform selective RARγ agonists and the single RARγ antagonist that are already available have opened entirely new phases to the therapeutic use of retinoids per se, with synthetic analogs offering horizon treatments for various diseases. This includes the prospect of killing cancer stem cells to provide perhaps a bona fide cure for some cancers. Molecular modeling and theoretical studies, supported by computer-assisted drug design, have an important role to play in getting new retinoids into the clinic to treat cancer and other targeted disorders.
